# A Systematic Review of Echocardiographic Parameters for the Screening of Pulmonary Hypertension: What Are the Odds?

**DOI:** 10.7759/cureus.32185

**Published:** 2022-12-04

**Authors:** Bathmapriya Balakrishnan, Bradley Owens, Ryan Hayes, Sijin Wen

**Affiliations:** 1 Pulmonary and Critical Care Medicine, West Virginia University, Morgantown, USA; 2 Department of Emergency Medicine, Allegheny Health Network, Pittsburgh, USA; 3 School of Medicine, West Virginia University, Morgantown, USA

**Keywords:** diagnostic odds ratio, likelihood ratio, systematic review, screening, pulmonary hypertension, echocardiography

## Abstract

Pulmonary hypertension (PH) is an insidious disease that often presents in late stages due to nonspecific signs and symptoms. Right heart catheterization (RHC) is the gold standard diagnostic test, and echocardiogram (ECHO) is the best screening tool. However, the strength of evidence and diagnostic utility of various echocardiographic parameters to screen for is not well elucidated. This systematic review (SR) is reported in accordance with the Preferred Reporting Items for Systematic Reviews and Meta-Analyses (PRISMA) statement. Literature searches was performed for the period of January 1, 2016, to June 1, 2021, on seven databases. We included full-text studies with adult patients that used RHC for comparison and provided sensitivity and specificity results. Likelihood ratios (LRs) and diagnostic odds ratios (DORs) were calculated. Risk of bias was assessed using the Quality Assessment Tool for the Observational Cohort and Cross-Sectional Studies. We identified 102 studies, but only 14 satisfied our inclusion criteria. The most significant parameters identified for PH screening based on LRs are, in descending order, tricuspid regurgitation gradient peak >36mmHg, systolic pulmonary artery pressure >41mmHg, and tricuspid regurgitation velocity >2.9 m/s. There is strong correlation between LR and DOR for these parameters. This SR indicates the superiority of some ECHO parameters over others to aid in the screening and severity assessment of PH. Variables with low LR (-) ratios may help to prevent unnecessary invasive assessment for PH. Clinicians should utilize a multi-parameter approach when interpreting echocardiograms for PH assessment.

## Introduction and background

Data from this study was presented at CHEST 2022 Annual Meeting on October 12-19, 2022, in Nashville, TN, USA. 

Pulmonary hypertension (PH) is an insidious disease that often presents in late stages due to nonspecific signs and symptoms. Most patients experience dyspnea on exertion, angina, fatigue, and occasionally syncope. Based on European Respiratory Society/European Society of Cardiologists (ERS/ESC) 2019 guidelines [1], PH is divided into five distinct categories by underlying pathophysiology leading to a common pathway of elevated mean pulmonary artery pressure (mPAP). The prevalence of the disease is difficult to measure in part because all five subcategories have significant variance [2]. The estimated prevalence is 10-52 cases per million [3]. Right heart catheterization (RHC) is the gold standard diagnostic test, and the less invasive echocardiogram (ECHO) is touted as the best screening tool to estimate pressures in the heart.

Rationale

While there have been multiple attempts to categorize ECHO parameters based on correlation with RHC, there have been relatively few systematic reviews (SR) examining the sensitivity, specificity, and likelihood ratios (LR) for individual parameters. There is no guidance provided on specific ECHO parameters and its corresponding evidence as compared to RHC to assess intracardiac pressures and right heart function to determine likelihood of PH or to track disease progression.

Objective

In this SR, we aim to establish the diagnostic utility of various ECHO parameters in the screening and severity assessment of PH. Our objective is to provide guidance to clinicians on when RHC is necessary to confirm the diagnosis and determine the severity of PH.

## Review

Methods

Source and Search

This SR is reported in accordance with the Preferred Reporting Items for Systematic Reviews and Meta-Analyses (PRISMA) statement [4]. Following the development of our study question, we performed our literature searches using subject heading terms and text words based on recognized and common indexing practices from January 1, 2016, to June 1, 2021, without language restrictions. The searches were limited to this period to capture new data in concert with recent advances in ECHO technology and to capture studies published after the updated PH guidelines were published in 2019. Search terms were compiled and tested repeatedly to increase sensitivity searches and to capture relevant publications. Literature searches were performed on these databases: PubMed, EbscoHost, Embase, Cochrane Review, Medline, Academic Search Complete, and Clinicaltrials.gov. Searches were performed independently by two investigators (RH and BO) using the following terms: pulmonary hypertension, ultrasound, diagnosis, echocardiography, ultrasonography, and point of care. Our search was supplemented by author and reference tracking for the identification of additional studies.

Selection

**Figure 1:**
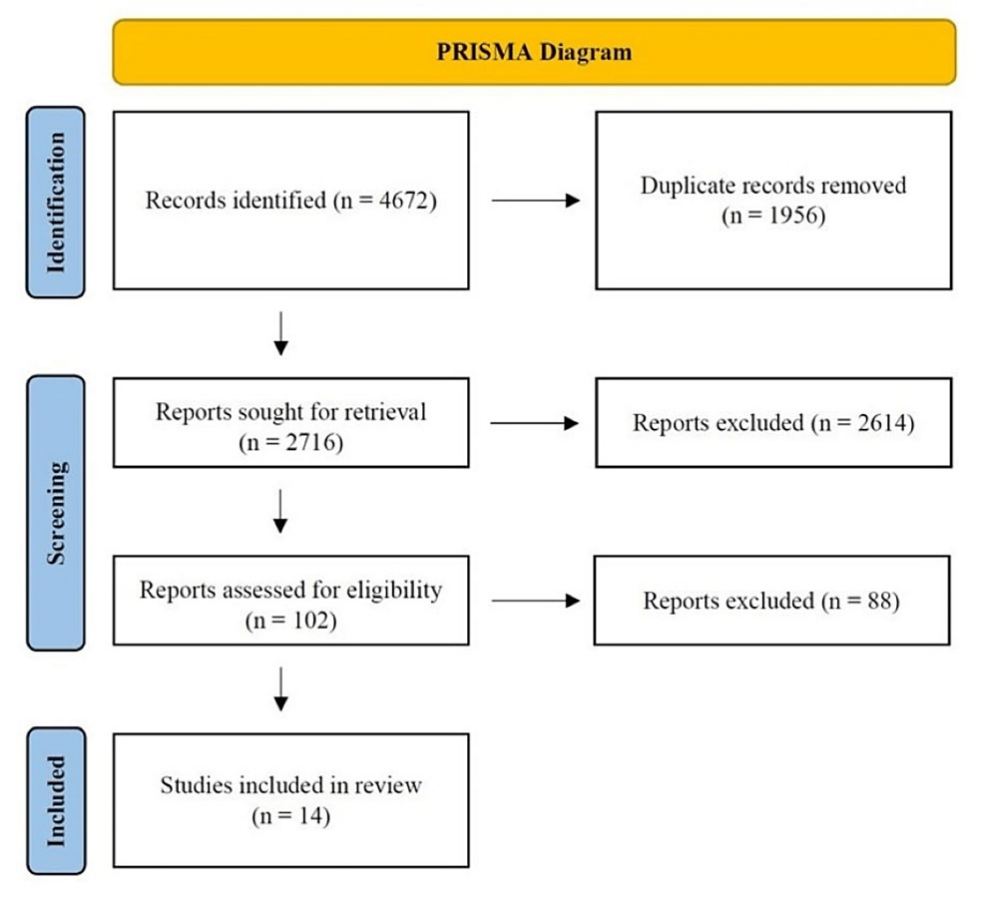
Preferred Reporting Items for Systematic Reviews and Meta-Analyses (PRISMA) Flow Diagram

We included full text studies following review of all titles and abstracts (Figure 1). Using EndNote, a web-based reference management software, we collected references in citation files, removed duplicates, and screened the studies using titles and abstracts. We excluded studies that did not meet our eligibility criteria and abstract-only studies. Abstracts were reviewed in full detail for inclusion in the full text screening phase. We included studies published in the English language that involved human adult patients, who were at least 18 years of age and diagnosed with PH via RHC. RHC is considered the gold standard tool to diagnose PH according to the 2019 ESC/ERS guidelines [1]. No cutoff value for RHC was established in our study because papers published during the study period included results before and after the 2019 ESC/ERS guidelines [1], which changed the definition of PH to a mPAP of 20mmHg from 25mmHg. Studies that did not compare ECHO to RHC findings were excluded. 

Data Extraction, Risk of Bias and Quality Assessment

**Figure 2:**
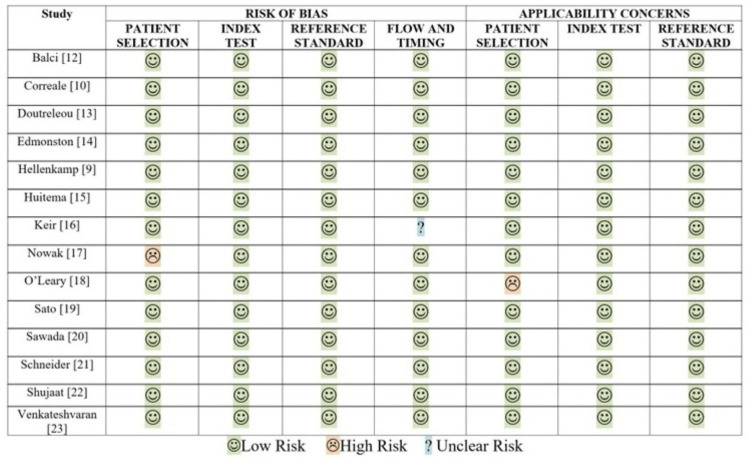
Quality and Bias Assessment

RH and BO reviewed each retrieved article, and all the data was extracted from full text articles, including tables and figures. Disagreements were addressed by consensus and by a third reviewer (BB). Data was extracted from studies, which included primary author, time of the study, year of publication, patient baseline characteristics, patient demographics, research setting, research methods, and diagnostic findings. Interobserver agreement for our study selection had a concordance of 0.89 and a kappa statistic 0.65. Assessment of the quality and risk of bias (Figure 2) of all included studies was independently performed by RH, BO, and BB employing the Quality Assessment Tool for the Observational Cohort and Cross-Sectional Studies available from the National Institute of Health [5] and the Cochrane Collaboration’s tool for assessing risk of bias, respectively [6].

Data Synthesis and Analysis

Pooled statistics were calculated where multiple studies examined identical parameters. Single study parameters are reported as they were in their respective papers. This SR identified 13 echocardiographic findings for comparison: tricuspid regurgitation peak gradient (TRG), tricuspid regurgitation velocity (TRV), tricuspid annular plane systolic excursion (TAPSE), mPAP, tricuspid regurgitation (TR) and right ventricular (RV) dilatation/dysfunction, left atrial diameter (LAD), pulmonary artery systolic pressure (PASP), right ventricular systolic pressure (RVSP), right ventricular outflow tract (RVOT) diameter, combination of RVOT and TAPSE, TR peak gradient, predictive equation (PE), and pulmonary vascular resistance (PVR). Using sensitivity and specificity, LRs and DORs were calculated for each variable (Table 1). DOR is an important tool in the analysis of tests studied by multiple experiments and describes the odds of a patient with disease experiencing a positive test [7].

**Table 1:** Echocardiographic Parameters *Correale et al. [10] DOR, Diagnostic odds ratio; LR, Likelihood ratio; mPAP, Mean pulmonary artery pressure; PASP, Pulmonary artery systolic pressure; PE, Predictive equation; PVR, Pulmonary vascular resistance; RV, Right ventricle; RVOT, Right ventricular outflow tract; RVSP, Right ventricular systolic pressure; Sen, Sensitivity; Sp, Specificity; TAPSE, Tricuspid annular plane systolic excursion; TR, Tricuspid regurgitation; TRG, Tricuspid regurgitation gradient peak; TRV, Tricuspid regurgitation velocity; WU, Woods unit.

Parameter	Sensitivity	Specificity	LR +	LR -	Positive Predictive Value	Negative Predictive Value	DOR
TR Peak >36mmHg	90	93	12.86	0.11	9.00	0.08	119.57
PASP >41mmHg	92	91	10.22	0.09	11.50	0.10	116.28
TRV >2.9 m/s	87	91	9.67	0.14	6.69	0.10	67.67
RVOT + TAPSE	100	83.3	5.99	0.00	0.00	0.20	0.00
RVOT Diameter >34mm	62.2	89.3	5.81	0.42	1.65	0.12	13.73
RVSP (>43mmHg)	92.3	81.8	5.07	0.09	11.99	0.22	53.88
mPAP (Aduen) pooled	92.41	75.65	3.80	0.10	12.18	0.32	37.83
Predictive equation* cutoff 57	93	67	2.82	0.10	13.29	0.49	26.97
PASP (>35mmHg)	85	67	2.58	0.22	5.67	0.49	11.51
TAPSE (<1.8cm) pooled	79.87	68.20	2.51	0.30	3.97	0.47	8.51
Mid RV Diameter (>2.8cm)	65.5	73.6	2.48	0.47	1.90	0.36	5.29
TRV >3.4 m/s pooled	57.17	75.35	2.32	0.57	1.33	0.33	4.08
PVR >3WU	81	65	2.31	0.29	4.26	0.54	7.92
TRV (>2.8m/s) pooled	59.21	73.09	2.20	0.56	1.45	0.37	3.94
TRV <2.8 /s	74	60	1.85	0.43	2.85	0.67	4.27
TAPSE (<1.7cm)	62.2	64.7	1.76	0.58	1.65	0.55	3.02
Left Atrial Diameter (>4.3cm)	62.4	63.4	1.70	0.59	1.66	0.58	2.87
TR + RV dilation/dysfunction	85	42	1.47	0.36	5.67	1.38	4.10
Absent TR	72	49	1.41	0.57	2.57	1.04	2.47
PASP >40mmHg	95	21.7	1.21	0.23	19.00	3.61	5.27
TRV <2.9 m/s	0	57	0.00	1.75	0.00	0.75	0.00

Quality Assessment

Our quality assessment and risk of bias are outlined in Figure 2. The quality of our included studies appears to be favorable. Results from the included studies show similar correlation between ECHO and RHC, and there is consistent homogeneity among similar parameters. Specifically, all patients in all the studies received an ECHO within a reasonable time interval in relation to the RHC. Many studies were retrospective, and the use of blinding was not reported. According to Oxford Centre for Evidence-based Medicine rating system, the quality of evidence from our included studies is a Level 2b [8]. Risk of bias was low. Selection bias was inherent as all patients were referred for RHC; many were from known or suspected PH cohorts or had previously known pulmonary disease. Performance bias was high since echocardiography is significantly dependent on user skill and experience. Observer bias was less likely, given the consecutive nature of many of the studies. Detection bias was indeterminable because many of the studies were retrospective in nature.

Results

Studies Selected and Characteristics

We identified 102 studies for abstract review. After discarding duplicates, we found 88 studies that qualified for full text review by two independent investigators (RH and BO). Fourteen studies met initial inclusion criteria for our SR (Figure 1). Study characteristics included in this review are summarized in Table 2. RHC was the gold standard for diagnosing PH in all our included trials.

**Table 2:** Study Characteristics *Correale et al. [10] ECHO, echocardiogram; ILD, interstitial lung disease; mPAP, mean pulmonary artery pressure; PASP, pulmonary artery systolic pressure; PH, pulmonary hypertension; PVR, pulmonary vascular resistance; RVOT, right ventricular outflow tract; RVSP, right ventricular systolic pressure; TAPSE, tricuspid annular plane systolic excursion; TR, tricuspid regurgitation; TRG, tricuspid regurgitation gradient peak; TRV, tricuspid regurgitation velocity.

Author	Publish Date	Study Type	Population	Study Group	Period	Median/mean Age (y)	% Female (%)	Parameter
Balci [12]	2016	Prospective Cohort	103	End stage lung disease transplant	1/12 - 9/12	48	34	PASP (>35mmHg)
Correale [10]	2019	Retrospective Cohort	64	Suspected PH	11/09-11/15	66	53	Predictive equation* cutoff 57
Doutreleau [13]	2016	Prospective Cohort	106	Suspected or known PH	5/11-6/12	65.6	49	mPAP (Aduen)
Edmonston [14]	2020	Retrospective Cohort	1714	Chronic Kidney Disease	1/11-12/14	69.1	46	TRV (>2.8m/s), TAPSE (<1.7cm), L Atrial Diameter (>4.3cm), and Mid RV Diameter (>2.8cm)
Hellenkamp [9]	2018	Retrospective Cohort	90	All RHC and Echo assessments	2011-2016	64.8	52.4	TRV (>2.8m/s), and mPAP (Aduen)
Huitema [15]	2020	Prospective Cohort	36	Sarcoidosis	8/15-11/18	57.6	27.8	TRV <2.9 m/s, and TRV >3.4 m/s
Keir [16]	2018	Retrospective Cohort	265	ILD	2006-2012	60.8	53.9	TRV <2.8 /s, TRV >2.8 m/s, TRV >3.4 m/s, and TR + RV dilation/dysfunction
Nowak [17]	2016	Retrospective Cohort	65	End stage lung disease transplant, pre-capillary PH	4/07-12/11	53.2	24.6	TAPSE (<1.8cm), RVSP (>43mmHg), RVOT Diameter >34mm, and RVOT + TAPSE
O'Leary [18]	2018	Retrospective Cohort	1262	All RHC patients	1998-2014	58.9	48.7	TRV (>2.8m/s), and Absent TR
Sato [19]	2017	Retrospective Cohort	54	Precapillary PH	4/10-2/13	56	75.9	TAPSE
Sawada [20]	2019	Retrospective Cohort	189	All RHC and Echo assessments	6/05-12/12	58	44	SPAP >41mmHg, and TR Peak >36mmHg
Schneider [21]	2017	Prospective Cohort	65	All RHC	7/15-7/16	67.2	57	TRV >2.9 m/s
Shujaat [22]	2016	Retrospective Cohort	87	All RHC and Echo assessments	7/08-6/12	54.3	79	TRV >2.8 m/s, and SPAP >40mmHg
Venketashan [23]	2019	Prospective Cohort	55	All RHC patients	2/14-2/18	58	33	PVR >3WU

Outcome

Pooled data for sensitivity and specificity were used to calculate DOR and LR, which are reported in Table 1. The most significant parameters identified are TRG > 36mmHg, SPAP > 41mmHg, TRV > 2.9 m/s, RVOT + TAPSE, RVOT diameter > 34mm, RVSP > 43mmHg, mPAP ≥ 25mmHg [9], and PE cutoff 57 as defined by Correale et al. [10]. These parameters demonstrated both positive and negative predictive value based on the results.

Discussion

General Interpretation

This SR focused on recent publications due to the improved quality of newer ultrasound devices and widespread use of ECHO for the evaluation of PH. RHC remains the gold standard for diagnosis, but this procedure is invasive and not without risks. Attempts to improve non-invasive screening have been ongoing since the 1980s. Despite recent updates to guidelines for the screening, diagnosis, and monitoring of PH, there is still not a consistently reliable method or parameter that would replace the need for invasive assessment. All the LRs were graded according to standard convention [11]. DORs are generally considered more likely to be positive if they are greater than one [7]. For echocardiographic LR values between 0 and 1 (negative LR), the lower the LR, the lower the probability of the patient screening positive for PH. Conversely, for echocardiographic LR values greater than 1 (positive LR), the higher the LR, the higher the probability of the patient screening positive for PH (Table 3) [11]. 

**Table 3:** Likelihood ratios and estimates of probability

Likelihood Ratio	Approximate Change in Probability (%)
0.1	-45
0.2	-30
0.3	-25
0.4	-20
0.5	-15
1	No change
2	+15
3	+20
4	+25
5	+30
6	+35
8	+40
10	+45

The data presented in this review have shown that certain ECHO parameters and combination of parameters have stronger diagnostic utility in relation to RHC to aid in the screening, diagnosis, and severity assessment of PH. Many individual parameters in the included studies had strong correlation with RHC, but they also had either poor sensitivity or poor specificity. Overall, most parameters studied had poor-to-fair diagnostic value by themselves. Parameters like TRG or RVOT + TAPSE had superior utility for ruling in PH because of high LR or DOR. Parameters such as SPAP, RVSP, or Correale’s PE with low LR or DOR may be useful in ruling out PH. LR or DOR of 0.1 decreases the odds of a condition being present by 45%. Thus, these parameters aids physicians in deciding to avoid unnecessary RHC. Using traditional sensitivity and specificity alone does not provide a clear picture, but the use of LR and DOR does improve the deterministic capability of a physician to pursue further invasive testing or to rule it out altogether in the assessment of PH.

Limitations of the Evidence

There were several limitations with the evidence used in this SR. Studies had significant variations for the same parameters despite similar overall strength. Multiple studies included highly specific subgroups of the populations such as lung transplant candidates, sarcoidosis, chronic kidney disease, or interstitial lung disease. Several papers focused on populations that were limited to specific ethnic groups or overrepresented a gender, which make the results less generalizable. Because of the populations included, a few studies had small sample groups, which likely overestimated the strength of their results. ECHO continues to be an operator-dependent imaging modality that requires significant practice and experience. Some of the included studies were forced to exclude patients with poor ECHO windows, which could represent a selection bias. Many of the patients included in numerous papers were in end-stage disease; this factor limits the generalizability of this SR to assess patients with early PH.

Limitations of the Review

There were a few limitations in our review process. Despite including papers published within the last five years, much of the data used in those papers was prior to the 2019 ERS/ESC updated guidelines [1]. This also meant that some of the papers used different cutoff values, which have since been abandoned. Included papers were limited to the English language. We only included papers that published or included sensitivity and specificity. Nearly all the papers included - and many which were excluded - reported correlation values, odds ratios, or positive/negative predictive values that may have significant research or diagnostic importance. Not all the parameters included in this paper were shared equally among the included papers, and many parameters reported were only found in a single paper.

Implications for Future Practice, Policy, and Research

This SR has identified several ECHO parameters that are superior to others in the screening of PH. ECHO parameters with low LR (-) ratios may help to prevent unnecessary invasive assessment for PH. Prospective studies conducted in patient populations with early PH should be pursued in the future. Additionally, a randomized controlled trial may be considered to validate the negative predictive value of some of these variables to prevent unnecessary invasive testing. Future studies should focus on established cutoff values to provide more research that is eligible for meta-analysis. With newer technology, it may be possible to combine echocardiography with either computed tomography or cardiac MRI, but further studies are needed to explore these modalities.

## Conclusions

This SR indicates the superiority of TRG and RVOT + TAPSE on ECHO to screen for PH based on the strength of LR and DOR. SPAP, RVSP and the PE are useful to rule out PH based on ECHO findings. While use of ECHO has grown exponentially in recent years, it is still insufficient to replace RHC for the diagnosis of PH. Coupled with clinical suspicion and patient history, use of these ECHO parameters can assist in the determination of referral for RHC. An SR or meta-analysis should be repeated in five to 10 years as more studies are completed assessing diagnostic utility of ECHO parameters using updated PH definitions.
